# Use of indirect evidence from HIV self-testing to inform the WHO hepatitis C self-testing recommendation

**DOI:** 10.1136/bmjgh-2022-011633

**Published:** 2023-02-14

**Authors:** Virginia A Fonner, Niklas Luhmann, Nandi Siegfried, Cheryl Johnson, Rachel Baggaley, Nathan Ford, Muhammad S Jamil

**Affiliations:** 1 Global Health and Population Research, FHI 360, Durham, North Carolina, USA; 2 Global HIV, Hepatitis and STI Programmes, World Health Organization, Geneva, Switzerland; 3 Independent Clinical Epidemiologist, Cape Town, South Africa

**Keywords:** Viral hepatitis, Health policy, Systematic review

**Summary box**

- The World Health Organization develops global normative guidelines using a comprehensive and transparent process involving critical evidence appraisals. Basing these appraisals on direct evidence alone may delay the introduction of new interventions, particularly for areas where indirect evidence exists from similar interventions or related health conditions.
- This study presents an example of application of indirect evidence from HIV self-testing to inform a recommendation for hepatitis C virus self-testing.
- This example supports pragmatic approaches for rapid development of global guidelines, such as situations where relevant and sufficient indirect evidence can facilitate accelerated introduction of new interventions to achieve public health impact.

## Introduction

Evidence appraisal is a critical component of global health normative guidelines development by the World Health Organization (WHO). Since 2007, WHO’s handbook for guidelines development has outlined a structured and transparent process to ensure guidelines are informed by the latest evidence on intervention effectiveness, feasibility of implementation and considerations on other important elements such as values and preferences of users and providers, resource use, equity and human rights.^1^ Typically, guidelines development involves a systematic review of evidence, appraisal of the certainty of evidence in accordance with the GRADE (Grading of Recommendations, Assessment, Development and Evaluations) framework^2^ and formulation of recommendations by an independent guidelines development group of experts. These guidelines groups typically comprise providers, academics, programme managers and affected populations with gender and geographical representation. Here, we present an example of a guidelines development process for a WHO recommendation on hepatitis C virus (HCV) self-testing with application of indirect evidence from HIV self-testing (HIVST).^3^ ^4^

Self-testing is a process whereby an individual collects their own specimen, performs a simple rapid test and interprets their own result. WHO first recommended HIVST in 2016.^5^ As of July 2022, 98 countries have national policies supportive of HIVST and 52 countries are routinely implementing HIVST to help achieve national and global goals.^6^ For HCV, despite recent advances in effective and affordable treatment, globally only 26% of the estimated 58 million people with chronic HCV infection were diagnosed as of 2021.^7^ New interventions and additional approaches, such as hepatitis C virus self-testing (HCVST), may help address this diagnosis gap so that more individuals can benefit from life-saving treatment.

To support the rapid development of WHO guidelines on HCVST, complementary sources of information were used, including: (1) a systematic review on the effectiveness of HCVST; (2) an update to a previous systematic review on the effectiveness of HIVST that informed the 2019 WHO recommendation on HIVST; (3) community-based HCVST usability and acceptability studies and community values and preferences; and (4) HCVST cost-effectiveness modelling. Below we outline the process and use of evidence sources with a focus on application of indirect evidence of intervention effectiveness in WHO guidelines development process.

## Main sources of evidence for HCVST guidelines development and key findings

### Systematic review on effectiveness of HCVST

We conducted a systematic review of the published and grey literature from 1 January 2010 to 16 December 2020. The review protocol was prospectively registered with PROSPERO (CRD42021235825). We included studies that compared HCVST to standard HCV testing services and measured one or more key outcomes, including: testing uptake; antibody positivity; linkage to additional testing following a reactive HCVST result; linkage to clinical assessment and/or treatment initiation among those diagnosed; proportion cured among those diagnosed; HCVST-related social harms, adverse events or misuse. We found no articles that met the inclusion criteria, thus no direct evidence on the effectiveness of HCVST was identified. Results of this review are detailed in an annex of the WHO HCVST guidelines.^4^

### Systematic review on effectiveness of HIVST

There was an *a priori* decision made in partnership with the guidelines group to include evidence on HIVST effectiveness given the similarity in intervention, diseases and affected populations. We used a living systematic review on HIVST for evidence,^8–10^ the methods for which are described elsewhere.^8^ Evidence from 27 randomized controlled trials (RCTs) showed that HIVST increased the uptake of testing among both key populations and the general population compared with standard facility-based testing. The proportion diagnosed HIV-positive and linked to treatment or care were similar but overall greater numbers of people were diagnosed and linked to care with HIVST. HIVST was acceptable to providers and users and reported HIVST-related harms, misuse or adverse events were rare.

### Community-based HCVST acceptability, usability and feasibility studies

WHO collaborated with the Foundation for Innovative Diagnostics to conduct HCVST usability studies among men who have sex with men, people who inject drugs and general populations across several countries. These studies showed that majority of participants were able to accurately perform and interpret their results using a prototype HCVST kit.^11 12^ A multicountry rapid qualitative assessment was also conducted to understand the values and preferences of potential users and providers.^13^ Overall, these rapid assessments found that HCVST was viewed positively across populations. The assessments also highlighted the need for further research on potential service delivery models for HCVST, given no real-world HCVST implementation had occurred at the time of the data collection.^13^

### Modelling on cost-effectiveness of HCVST

A cost-effectiveness modelling study was conducted to identify drivers of cost of testing or HCV cure with introduction of HCVST across four countries.^14^ The cost-effectiveness model suggested that HCVST is likely to be costlier than standard testing but will reach more people for testing.^14^

## Utility of using indirect evidence

The systematic review identified no direct evidence on HCVST effectiveness. However, extensive indirect evidence on HIVST found the intervention to be safe, effective, acceptable and feasible across a range of populations and settings. Extensive implementation experiences now exist across the globe that can inform routine implementation. Additional direct, supportive evidence showed HCVST is perceived as beneficial and acceptable by potential users and providers and may identify additional infections. Taken the available sources of evidence together, the guidelines group, by consensus, determined that HCVST should be offered as an additional approach to HCV testing as a WHO recommendation.^4^

This novel approach can expedite the guideline development for new interventions where evidence from similar interventions exists with sufficient overlap in epidemic contexts (eg, health condition characteristics, populations at-risk and programming), with important caveats. We purposefully have not further defined these criteria as each requires careful consideration and specification according to the intervention under consideration. For example, defining what level of population overlap and within which characteristics will be a case-specific determination. Additionally, there are some instances where sole use of indirect evidence would never be appropriate, such as for drugs or vaccines that would require specific safety and efficacy evaluations among the populations being considered. The specific example presented in this commentary—HCVST—relates to a testing approach for an infectious disease—a decidedly different type of intervention as compared with a pharmacological treatment or vaccine. Given the rise of differentiated service delivery models and self-care approaches within a growing number of health areas (eg, HIV, sexually transmitted infections, COVID-19 and family planning), one could envision taking an approach to guideline development using indirect evidence within these intervention types when feasible. For HIV, hepatitis, tuberculosis and sexually transmitted infections, integrated service delivery models are already being implemented or considered.

Additionally, when using indirect evidence, it is important to note differences that exist across the intervention under consideration and the comparator. For example, for the HCVST guidelines, no HIVST trial was identified among people who inject drugs—a key priority population for HCV. Thus, this population was purposefully prioritised for usability studies. There are also differences in HCV and HIV testing (ie, serological tests for HIV are sufficient for diagnosis, which is not the case for HCV). Additionally, no evidence on HCV cure rates was available as it is not applicable to HIV. An a priori decision was thus taken to use linkage to HIV treatment or care among those diagnosed HIV-positive as a proxy for potential cure rates. The guidelines group convened by WHO noted sufficient similarity between HIVST and HCVST in terms of interventions, programming and priority populations and deemed that indirect HIVST evidence would be applicable to HCVST. The guidelines group also identified research gaps and priorities that would inform future implementation considerations, including the need for more HCVST research among people who inject drugs, innovative service delivery models and efficient referral pathways.

## Conclusions

In this commentary, we described a pragmatic guideline development process that occurred with available indirect and supportive evidence in the absence of direct evidence. As evidence from RCTs for new interventions can take several years to generate, this process accelerated the recommendation of HCVST, thus potentially accelerating improved health outcomes among affected populations. For other urgent public health problems, such processes for guideline development can potentially expedite availability of interventions that have proved effective in similar contexts. Developing more specific, standardised procedures and criteria for determining when the application of indirect evidence is appropriate are needed, and we welcome engagement to further refine this.

Basing recommendations on mostly indirect evidence necessitates close monitoring of implementation and highlights a need to address research gaps, such as understanding the feasibility of implementation among certain populations, within certain contexts, or using certain delivery approaches that were not included in the evidence used to inform the initial recommendation.

Results from the HCVST guidelines process provide a potential roadmap for navigating this process in situations where direct evidence is lacking but indirect evidence is available and appropriate to use. In such instances, the guideline development process can be made more efficient through use of accessible resources and information sharing among guideline developers. Additionally, obtaining feedback from stakeholders across the relevant interventions is important to ensure consensus with using indirect evidence to inform policy. WHO plans to replicate this process for other similar conditions such as syphilis self-testing.

## Data Availability

Data referenced in this article are publicly available.
